# Computational and Comparative In Vitro Evaluation of GC–MS Profiled *Cannabis sativa* Inflorescence Extracts’ Metabolites on Selected Diabetes Therapeutic Targets

**DOI:** 10.1002/cbdv.71684

**Published:** 2026-09-20

**Authors:** Rosa M. Chinheya, Viviane L. N. Ngoungoure, Ibeabuchi Ali, Kolawole Olofinsan, Tsebo Jim, Francine Tankeu, Motlalepula G. Matsabisa

**Affiliations:** ^1^ African Medicines Innovations and Technologies Development (AMIDT) Unit Department of Pharmacology University of the Free State Bloemfontein South Africa; ^2^ Department of Biochemistry Faculty of Science University of Yaoundé 1 Yaoundé Cameroon; ^3^ Department of Pharmaceutical and Medicinal Chemistry Faculty of Pharmaceutical Sciences Enugu State University of Science and Technology Enugu Nigeria

**Keywords:** antidiabetic protein targets, antioxidants, cannabinoids, *Cannabis sativa*, inflorescence

## Abstract

*Cannabis sativa*, a medicinal plant rich in cannabinoids, terpenoids, and flavonoids, has been shown to have various pharmacological activities. This study investigated the antidiabetic potential of *C. sativa* inflorescence extracts using in vitro and computational models. Dried *C. sativa* inflorescences were extracted sequentially with hexane, dichloromethane, and methanol, before their metabolites were identified by GC–MS. The extracts were tested for α‐amylase and α‐glucosidase inhibition, glucose uptake activity, and antioxidant effects using DPPH and nitric oxide (NO) inhibition assays. Thirty‐six metabolites were further docked against diabetes‐related proteins. Among the extracts, the hexane extract showed the strongest bioactivity with notable α‐amylase inhibition (IC_50_: 727 µg/mL), DPPH free radical scavenging (IC_50_: 478.62 µg/mL), and nitric oxide inhibition activity (IC_50_: 356.51 µg/mL). Docking analyses revealed strong binding affinities for 8‐hydroxy‐delta‐9‐THC, cannabivarin, and 9‐tetrahydrocannabinol with DPP‐4, PTP1B, and other target proteins. These findings highlight the potential of *C. sativa* inflorescences as a source of antidiabetic agents, warranting further in vivo and *clinical* validation.

## Graphical abstract

*Cannabis sativa* inflorescence extracts demonstrate promising antidiabetic potential through enzyme inhibition, antioxidant activity, and molecular docking. The hexane extract exhibits the strongest bioactivity, while key cannabinoids show high affinity for diabetes‐related targets, highlighting the plant as a promising source of novel antidiabetic compounds.

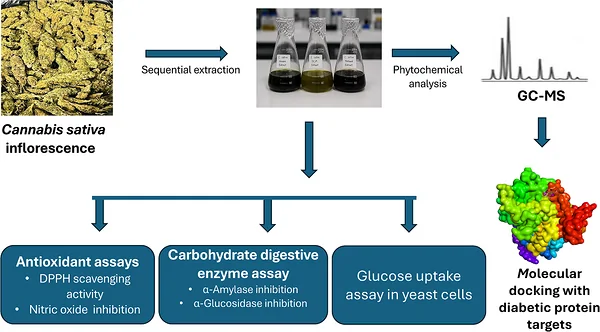

## 1 Introduction

*Diabetes mellitus* (DM) is a chronic metabolic disease characterized by elevated blood glucose (blood sugar) levels, which may be associated with abnormal insulin activity. Insulin is a pancreatic hormone that regulates blood sugar levels by promoting the absorption of glucose into cells for the generation and storage of energy [1]. The majority of people with diabetic conditions around the world have Type 2 diabetes, which is caused mainly by being overweight and a lack of physical activity, coupled with the body's inability to use insulin effectively. It has been scientifically proven that untreated or undiagnosed diabetes is associated with failure of body organs and tissues such as the heart, blood vessels, eyes, kidneys, and nerves, posing a serious health challenge and with an ultimate economic burden, especially in developing countries, due to lifestyle changes, as reported by Nwafor, et al. [2]. The International Diabetes Federation (IDF) reported that over 600 million people worldwide are living with diabetes, with the majority living in underdeveloped and developing countries. Due to the economic, social, and health background of people living in developing countries, the majority of the people living with diabetes are not receiving appropriate treatment. There has been a steady increase in both the number of people with diabetes and the number of those living with untreated diabetes over the past years [2]. Most therapeutic approaches for diabetes aim to reduce postprandial hyperglycemia by inhibiting α‐amylase and α‐glucosidase, carbohydrate‐hydrolyzing enzymes. Postprandial hyperglycemia is characterized by hyperglycemic spikes that induce endothelial dysfunction, inflammatory responses, and oxidative stress [3, 4].

Several molecular targets have been explored for the management of type 2 DM, each acting through distinct mechanisms to improve glucose homeostasis. α‐Amylase and α‐glucosidase are digestive enzymes that hydrolyze carbohydrates into simple sugars that can be absorbed into the bloodstream. The inhibition of this enzyme therefore delays glucose absorption, thereby reducing postprandial hyperglycaemia. Besides these carbohydrate‐digesting enzymes, proteins such as sodium‐glucose cotransporter‐2 (SGLT‐2), which mediates renal glucose reabsorption; dipeptidyl peptidase‐4 (DPP‐4), which regulates incretin hormone degradation; and protein tyrosine phosphatase 1B (PTP1B), which negatively regulates insulin signaling, are other targets for antidiabetic drugs. While the inhibition of SGLT‐2 increases glucose excretion in the kidney, similar action on DPP‐4 and PTP1B ultimately enhances insulin secretion in the pancreas. Some conventional drugs currently used to treat diabetes are derived from natural products. For instance, metformin and acarbose were derived from *Galega officinalis L*. and Actinoplanes spp., respectively [5, 6]. Although many commercially available chemical antidiabetic drugs have demonstrated some level of effectiveness in diabetes management, their affordability remains a major challenge, especially for patients in many rural sub‐Saharan African regions, necessitating the exploration of natural plant‐based alternatives [7, 8].

*Cannabis sativa* L. is a dioecious plant that belongs to the *Cannabaceae* family. It is widely used in traditional medicine to manage various conditions, including arthritis, diabetes, pain, cancer, skin disorders, gastrointestinal disorders, tuberculosis, and cough [9]. Different parts of *C. sativa* (leaves, roots, flowers, and whole plants) have been scientifically investigated using various preclinical, in vitro, in vivo, and cell‐based bioassay models. Notably, *C. sativa* demonstrated reasonable pharmacological activities, such as analgesic, anxiolytic, anticonvulsant, antidepressant, antihypertensive, anti‐inflammatory, antimicrobial, antioxidant, antidiabetic, antiproliferative, antitumor, cytotoxic, expectorant, and gastroprotective effects [10]. These pharmacological effects exhibited by *C. sativa* extracts, fractions, or isolates are attributed to copious bioactive metabolites, including cannabinoids, the major group of bioactive constituents, as well as flavonoids, terpenoids, and others [11]. Bioactive secondary metabolites have been implicated in attenuating the development and progression of diabetic conditions. They have shown potential as therapeutic agents in the management of diabetes due to their capacity to reduce oxidative stress, suppress pro‐inflammatory cytokines, and inhibit enzymes (α‐glucosidase and α‐amylase) involved in the digestion and absorption of carbohydrates [12]. Considering the polarity of bioactive secondary metabolites and their role in alleviating physiological disorders and eliciting therapeutic effects, the organic solvent used to extract *C. sativa* should be optimized to obtain an extract with the highest antidiabetic activity. Therefore, the present research seeks to compare the in vitro antidiabetic activities (α‐glucosidase, α‐amylase, and glucose uptake) of different solvent extracts of *C. sativa* inflorescence.

## 2 Materials and Methods

### 2.1 Chemicals and Reagents

Hexane, dichloromethane, and methanol solvents were purchased from Glassworld, South Africa. 1,1‐Diphenyl‐2‐picrylhydrazyl (DPPH), sodium nitroprusside, sulfanilamide, N‐(1‐naphthyl) ethylenediamine dihydrochloride, orthophosphoric acid, sodium hydroxide, ibuprofen, gallic aid, dimethyl sulfoxide (DMSO), sodium potassium tartrate tetrahydrate, Hydrochloric acid (HCl), ascorbic acid, starch, glucose, 3,5‐dinitrosalicylic acid (DNSA), acarbose, α‐amylase from porcine pancreas, α‐glucosidase from saccharomyces cerevisiae, 4‐nitrophenyl β‐D‐glucopyranoside, Silica gel 60 RP‐18 F_254_S aluminum plates were purchased from Sigma‐Aldrich, South Africa. Commercial baker's yeast was purchased from the local supermarket.

### 2.2 Collection and Processing of Plant Material

The plant material used was from a single batch of 100 kg of *C. sativa* inflorescence collected in the Lusikisiki area of the Eastern Cape Province of South Africa (30.9513°S, 29.83475°E) at an altitude of about 687m above sea level in September 2025. The authorization permit (POS No: POS 281/2025/2026) for handling, transportation, possession, and storage of cannabis for research purposes was obtained from the South African Health Products Regulatory Authority (SAHPRA). The plant was authenticated by “Geo Potts Herbarium” at the University of the Free State, Bloemfontein, South Africa, with a voucher specimen number BLFU MGM 0018. The *C. sativa* inflorescences were dried at room temperature in the laboratory extraction room of the AMITD, Department of Pharmacology, University of the Free State. After about 2 weeks of air‐drying, the material was pulverized into fine powder using a benchtop hammer mill.

### 2.3 *C. sativa* Inflorescence Extraction

Phytochemicals were exhaustively extracted from the plant material using three solvents of increasing polarity (hexane, dichloromethane, and methanol). A plant‐to‐solvent ratio of 1:5 (w/v) was employed to ensure adequate solvent penetration and efficient mass transfer during extraction. The mixtures were extracted at ambient room temperature with constant agitation on an FMH 200 shaker at 155 rpm and filtered using a Rousselet centrifuge at 3000 rpm. Extraction with each solvent continued until the filtrate became colorless, indicating exhaustive extraction of soluble constituents. The extracts were concentrated using a BUCHI Rotavapor R‐300 with the water bath set at 40°C. The concentrated extracts were then dried at room temperature in labelled containers, in a fume hood, before storage at 4°C until further use.

### 2.4 GC–MS Profiling of the Inflorescence Extracts

The samples were dissolved in 1 mL of the extraction solvent. Analysis was carried out with a Thermo Scientific Trace 1310 gas chromatograph fitted with a flame ionization detector (FID), and an SGE BP5MS analytical column of 60 m x 0.25 mm x 0.25 µm (length x inner diameter x film thickness) coupled to a Thermo Scientific ISQ 7000 single quadrupole mass spectrometer (MS). Hydrogen was used as a carrier gas at a pressure of 200 kPa. The injector and FID were maintained at 290°C, the ion source at 200°C, and the transfer line at 280°C. The MS scan range was 50–600 *m/z*. The initial oven temperature was set at 60°C for 10 min, then increased at a rate of 5°C/minute to 300°C, where it was maintained for 30 min. Instrument control and data analysis were performed using Thermo Xcalibur 4.0 software, and tentative compound assignments were based on comparison of the acquired mass spectra with entries in the 2017 NIST Mass Spectral Library.

### 2.5 Thin Layer Chromatography

Thin layer chromatography (TLC) was performed on silica gel 60 RP‐18 F_254_S aluminum plates (Merck, Germany). Extracts and standard cannabinoids (CBD, CBN, CBG, and CBC) were spotted and developed in a mobile phase consisting of methanol: water (9:1, v/v) for 10 min. After air‐drying, developed plates were visualized under UV light at 254 nm. Retention factor (Rf) values were calculated.

### 2.6 α‐Amylase Inhibition Assay

The ability of the extracts to inhibit α‐amylase enzyme was determined using the procedure described by Kifle et al. [13] with slight modifications. Each extract was initially dissolved in DMSO before further dilution with sodium phosphate buffer to the desired concentrations. The final concentration of DMSO in the 500 µg/mL test concentration was 0.5% (v/v). Then, 100 µL of each extract concentration (62.5, 125, 250, and 500 µg/mL) and acarbose (100 µL) at various concentrations 12.5, 25, 50, and 100 µg/mL were added to 100 µL of α‐amylase solution (5 mg/mL) and incubated in a water bath for 10 min at 37°C. Subsequently, 100 µL of starch solution (1%) was added to the mixture and incubated at 37°C for 30 min in a water bath. Thereafter, 200 µL of DNSA was added to the mixture, and the reaction mixture was boiled for 5 min. After cooling in an ice bath, 40 µL of each reaction mixture was transferred to a 96‐well plate and diluted with 210 µL of distilled water. Absorbance was measured at 540 nm. For all colorimetric assays, a corresponding sample blank was prepared for each extract concentration to account for the intrinsic color of the plant extracts and the solvent background. The absorbance of the sample blank was subtracted from the measured sample absorbance prior to calculating the percentage inhibition. The percentage inhibition of each sample was calculated against a control experiment containing 0.5% DMSO in sodium phosphate buffer, instead of the test samples, using the following formula:

(1) $$\%\mathrm{Inhibition}\;=\frac{\mathrm{Control}\;\mathrm{absorbance}\;-\mathrm{Sample}\;\mathrm{absorbance}}{\;\mathrm{Control}\;\mathrm{absorbance}\;}\;\times 100$$

### 2.7 α‐Glucosidase Inhibition Assay

α‐Glucosidase inhibitory capacity of the extracts was analyzed according to the procedure described by Mnge et al. [14] with minor modifications. Each extract was initially dissolved in DMSO before further dilution with sodium phosphate buffer to the desired concentrations. The final concentration of DMSO in the 500 µg/mL test concentration was 0.5% (v/v). Then, 120 µL of each extract solution at various concentrations (62.5, 125, 250, and 500 µg/mL) or acarbose, used as the standard at different concentrations 0.125, 0.25, 0.5, and 1 mg/mL, was added to 20 µL of α‐glucosidase solution (0.5 U/mL) in a 96‐well plate. The plate was then incubated at 37°C for 15 min. Subsequently, 30 µL of 4‐nitrophenyl β‐D‐glucopyranoside substrate (5 mM) was added to the mixture and incubated for another 15 mins at 37°C. The reaction was stopped by adding 80 µL of 0.2 M Na_2_CO_3._ Absorbance was measured at 415 nm, and the percentage inhibition of each sample was calculated against a control test solution containing 0.05% DMSO and buffer instead of the test samples using the same Equation (1) as previously described.

### 2.8 Glucose Uptake in Yeast Cells

Glucose uptake in yeast cells was carried out based on the method described by Aloysius et al. [15] with slight modifications. Commercial baker's yeast was dissolved in distilled water to prepare a 1% suspension and incubated overnight at 37°C. After 24 h, the yeast cell suspension was centrifuged at 3500 rpm for 5 min. The centrifugation step was repeated by resuspending the pellet in distilled water until a transparent supernatant was obtained. Then, 10 mL of the supernatant was mixed with 90 mL of distilled water to get the yeast cell suspension. Extracts at various concentrations (62.5, 125, 250, and 500 µg/mL) were incubated with glucose solution (50 mM) for 10 min at 37°C. The reaction was initiated by adding 100 µL of yeast suspension to the samples of glucose and extracts. The samples were vortexed and incubated for one more hour at 37°C. After the incubation, 3,5‐dinitrosalicylic acid (DNSA) was added to the tubes, and was placed in boiling water for five minutes without allowing the tubes to boil. The glucose uptake was read at 540 nm. Absorbance for the control was carried out on a similar wavelength. The percentage increase in glucose uptake was calculated using the formula:

$$\begin{array}{ccc} & & \%\;\mathrm{Increase}\;\mathrm{in}\;\mathrm{glucose}\;\mathrm{uptake}\;\\ & & \;=\frac{\mathrm{Control}\;\mathrm{absorbance}\;-\mathrm{Sample}\;\mathrm{absorbance}}{\;\mathrm{Control}\;\mathrm{absorbance}\;}\;\times 100. \end{array}$$

### 2.9 In Vitro 2,2‐Diphenyl‐1‐Picrylhydrazyl (DPPH) Scavenging Assay

DPPH scavenging capacity of the extracts was determined according to the method described by Nkemzi et al. [16] with some modifications. Extracts at various concentrations (62.5–500 µg/mL) were prepared, and 40 µL of each dilution was transferred into a 96‐well plate in triplicate. Subsequently, 160 µL of freshly prepared DPPH solution (0.004% DPPH dissolved in methanol) was added to each well. The mixture was incubated in the dark for 30 min, and the absorbance was measured at 517 nm using a microplate reader. Ascorbic acid was used as a standard, and all measurements were recorded. The percentage of inhibition was determined using formula (1) described previously.

### 2.10 In Vitro Nitric Oxide Inhibition Assay

Nitric oxide (NO) was generated from sodium nitroprusside and measured using the Griess reaction, as described by Pradubyat et al. [17] with minor modifications. Sodium nitroprusside in aqueous solution at physiological pH spontaneously generates NO, which interacts with oxygen to produce nitrite ions. These ions can be quantified using the Griess reagent. For the assay, sodium nitroprusside (10 mM) in phosphate‐buffered saline (pH 7.4) was mixed with different concentrations (62.5–500 µg/mL) of each extract and incubated at 30°C for 2 h. A reaction mixture containing the same volume of PBS without the extract served as the control. After the incubation period, Griess reagent (1% sulfanilamide, 2% orthophosphoric acid, and 0.1% N‐(1‐naphthyl) ethylenediamine dihydrochloride) was added for nitrite detection. The absorbance of the chromophore formed during diazotization of the nitrite was immediately read at 540 nm. The inhibition of nitrite formation by the plant extracts and the standard ibuprofen was calculated relative to the control.

### 2.11 Molecular Docking of GC–MS Identified Compounds

To explore the potential interactions between the identified phytochemicals and selected diabetes‐related protein targets, molecular docking was performed as an exploratory computational approach. An in‐house library of 36 compounds from different extracts and 4 standard inhibitor chemicals was subjected to molecular docking with selected antidiabetic protein targets. These latter macromolecules included α‐amylase and α‐glucosidase tested in this study, as well as dipeptidyl peptidase‐4 (DPP‐4), sodium glucose co‐transporter 2 (SGLT‐2), and protein tyrosine phosphatase 1B inhibitor (PTP1B). The 3D chemical structures of the compiled inflorescence compounds and respective chemical inhibitors of the proteins, including acarbose, sitagliptin, empagliflozin, and 3,4‐dibromo‐5‐(2‐bromo‐3,4‐dihydroxy‐6‐(isopropoxymethyl)benzyl)benzene‐1,2‐diol [18], were retrieved in SDF file format from the PubChem database. The structures were imported one after the other into the working directory of UCFS Chimera and then prepared for docking with the DockPrep tool of the software. Each of the output files was then saved in the mol2 file format into a desktop folder. Similar structural preparation, which includes removal of nonstandard atoms and co‐crystallized water molecules, followed by addition of polar hydrogen and Gasteiger charges, was done on 3D structures of the target proteins retrieved from the Protein Data Bank (PDB) website. The PDB identity numbers of the proteins are 1B2Y (α‐amylase), 3I4W (α‐glucosidase), 4A5S (DPP‐4), 7VSI (SGLT‐2), and 4Y14 (PTP1B). The gradient‐based local search genetic algorithm of AutoDock Vina in the UCSF Chimera software was then employed to conduct molecular docking of the compounds with the proteins. Subsequently, Discovery Studio software was used to generate 2D molecular interactions of the best binding pose of each compound with the respective proteins.

### 2.12 Statistical Analysis

Each experiment was performed independently on three separate occasions, with each independent experiment conducted in three technical replicates (*n* = 3), and the final presented data are expressed as mean ± standard deviation (SD). One‐way analysis of variance was conducted at the 0.05 level of significance. This statistical step was followed by Tukey's multiple comparison test between the different treatments at each concentration. The IC_50_ values were estimated using a linear regression analysis of mean activity against the log_10_ of concentrations in Microsoft Excel 365. While this software was also used for data preprocessing and analysis, data presentation was done using GraphPad Prism V6.

## 3 Results

### 3.1 Percentage Yield of the *C. sativa* Inflorescence Extracts

After drying, the percentage yield of the various concentrated extracts obtained from the studied plant was calculated. The most nonpolar hexane extract employed first produced an extract yield of 16.3%. The DCM solvent employed thereafter gave 3.16% extract yield, whereas the last polar methanol solvent generated a yield of 2.93%.

### 3.2 Bioactive Constituents in *C. sativa* Inflorescence Extracts

The GC–MS chromatogram and mass spectra obtained in positive ion mode from NIST library data were used to tentatively identify bioactive constituents of the hexane extract (Table 1). The following class of compound were revealed as cannabinoids, including Δ9‐tetrahydrocannabivarin, cannabivarin, cannabidiol, cannabinol, tetrahydrocannabinol (THC), and cannabigerol, with retention times (RT) between 50.69–57.21 min. Terpenes, including caryophyllene, humulene, eudesma‐4(14),11‐diene, and elina‐3,7(11)‐diene, were detected at RT 30.19–33.61 min, while fatty acid esters such as hexadecanoic acid ethyl ester (palmitate) and octadecanoic acid ethyl ester (stearate) were observed at RT 43.67–47.62 min. A steroid, 11‐ketoprogesterone, was detected at RT of 53.12 min.

**TABLE 1:** Phytochemicals present in *C. sativa* inflorescence hexane extract.

S/N	Retention time	Name of compound	Molecular weight	Fragment ion *m/z*	Formula
1	30.19	Caryophyllene	204	56,69,93,133,161,189,204	C_15_H_24_
2	31.24	Humulene	204	80,93,121,204	C_15_H_24_
3	32.22	Eudesma‐4(14),11‐diene	204	41,105,121,161,204	C_15_H_24_
4	33.61	Selina‐3,7(11)‐diene	204	91,122,161,204	C_15_H_24_
5	34.84	Caryophyllene oxide	220	43,79,93,95,121,135,177,220	C_15_H_24_O
6	35.07	5‐Azulenemethanol	222	59,107,161,163,204,222	C_15_H_26_O
7	35.58	Humulene epoxide I	220	67,109,138,159,220	C_15_H_24_O
8	36.16	Aromadendrene oxide	220	55,59,67,105,189,220	C_15_H_24_O
9	37.21	Bisabolol	222	69,109,119,121,204,222	C_15_H_26_O
10	43.67	Hexadecanoic acid ethyl ester	284	43,55,88,101,157,239,284	C_18_H_36_O_2_
11	47.09	9,12‐Octadecadienoic acid, ethyl ester	308	67,81,95,96,109,136,308	C_20_H_36_O_2_
12	50.69	Tetrahydrocannabivarin	286	41,43,81,91,203,271,286	C_19_H_26_O_2_
13	51.12	Cannabivarin	282	55,238,267,282	C_19_H_22_O_2_
14	52.44	Cannabidiol	314	121,174,231,232,314	C_21_H_30_O_2_
15	53.12	11‐Ketoprogesterone	328	43,91,122,147,207,241,328	C_21_H_28_O_3_
16	54.44	Tetrahydrocannabinol	314	55,91,115,128,231,271,299,314	C_21_H_30_O_2_
17	55.16	Cannabigerol	316	123,193,231,247,316	C_21_H_32_O_2_
18	55.55	Cannabinol	310	43,119,165,238,295,296,310	C_21_H_26_O_2_

*Note*: Compounds were tentatively identified using Thermo Xcalibur 4.0 software by comparing mass spectra with those in the 2017 NIST Mass Spectral Library, and the confidence level for molecular identification was based on NIST library matches.

The GC–MS chromatogram and mass spectra obtained in positive ion mode from NIST library data were used to tentatively identify bioactive constituents of the dichloromethane extract. As presented in Table 2, 17 compounds with retention times ranging from 30.18 to 57.20 min. Cannabinoids, including tetrahydrocannabivarin, cannabidiol, tetrahydrocannabinol, cannabigerol and cannabinol, were detected at retention times between 50.69 and 57.20 min. Fatty acids and esters comprised of cis‐5,8,11,14,17‐eicosapentaenoic acid (37.21 min), pentadecanoic acid methyl ester (42.28 min), hexadecanoic acid ethyl ester (43.66 min), and ethyl‐9,12‐octadecadienoate (47.08 min). Terpenes identified included caryophyllene (30.18 min), limonene (32.73 min), caryophyllene oxide (34.84 min), eudesm‐4(14)‐en‐11‐ol (36.73 min), and neophytadiene (40.19 min). Additional compounds detected were 1,1,4,4,7,7‐hexamethyltrindan (51.83 min) and 1‐chloro‐3,6‐dimethoxycrinan‐2‐ol (52.72 min).

**TABLE 2:** Phytochemicals present in *C. sativa* inflorescence DCM extract.

S/N	Retention time	Name of compound	Molecular weight	Fragment ion *m/z*	Formula
1	30.18	Caryophyllene	204	93, 121, 147,204	C_15_H_24_
2	32.73	Limonene	184	67,69,91,95,98,166,184	C_10_H_16_O_3_
3	34.84	Caryophyllene oxide	220	56,79,93,95,109,136,177,220	C_15_H_24_O
4	36.73	Eudesm‐4(14)‐en‐11‐ol	222	43,59,108,149,164,189,204,222	C_15_H_26_O
5	37.21	Cis‐5,8,11,14,17‐Eicosapentaenoic acid	302	79,91,93,119,133,175,292,302	C_20_H_30_O_2_
6	40.19	Neophytadiene	278	68,82,95,123,124,137,193,278	C_20_H_38_
7	41.15	2‐Hexadecen‐1‐ol 3,7,11,15‐tetramethyl	296	82,95,123,124,279,296	C_20_H_40_O
8	42.28	Pentadecanoic acid 14‐methyl‐ methyl ester	270	74,87,143,270	C_17_H_34_O_2_
9	43.66	Hexadecanoic acid, ethyl ester	284	43,53,88,107,157,239,284	C_18_H_36_O_2_
10	47.08	Ethyl‐9,12‐octadecadienoate	308	67,81,95,96,110,164,308	C_20_H_36_O_2_
11	50.69	Tetrahydrocannabivarin	286	41,43,81,189,203,271,286	C_19_H_26_O_2_
12	51.83	1,1,4,4,7,7‐Hexamethyltrindan	282	55,238,267,282	C_21_H_30_
13	52.43	Cannabidiol	314	68,121,193,231,314	C_21_H_30_O_2_
14	52.72	1‐Chloro‐3,6‐dimethoxycrinan‐2‐ol	367	42,56,77,217,332,367	C_18_H_22_ClNO_5_
15	54.39	Tetrahydrocannabinol‐7‐oic acid	314	41,91,174,231,271,299,314	C_21_H_30_O_2_
16	55.15	Cannabigerol	316	69,123,193,231,316	C_21_H_32_O_2_
17	57.20	Cannabinol	310	57,211,254,295,310	C_21_H_26_O_2_

*Note*: Compounds were tentatively identified using Thermo Xcalibur 4.0 software by comparing mass spectra with those in the 2017 NIST Mass Spectral Library, and the confidence level for molecular identification was based on NIST library matches.

Moreover, in Table 3, twenty‐one (21) compounds were tentatively identified using GC–MS chromatogram and mass spectra obtained in positive ion mode from NIST library data. The following compounds were present in the methanol extract Cannabinoids, (tetrahydrocannabinol, tetrahydrocannabivarin, cannabivarin, cannabidiol, tetrahydrocannabinol, cannabigerol, cannabinol, and 8‐Hydroxy‐delta‐9‐THC, were detected at 49.98, 50.69, 51.81, 52.43, 54.44, 55.14, 55.51, and 56.73 min retention times, respectively), Fatty acids, esters, and long‐chain alcohols (hexadecanoic acid ethyl ester (43.66 min), 9,12‐octadecadienoic acid ethyl ester (47.01 min), 1‐heptatriacotanol (47.96 min), and hexadecane, 1,1‐bis(dodecyloxy)‐ (43.28 min)) and terpenes (caryophyllene (30.18 min), 7‐epi‐trans‐sesquisabinene hydrate (33.84 min), caryophyllene oxide (34.84 min), 2‐(4a,8‐dimethyl‐2,3,4,5,6,8a‐hexahydro‐1*H*‐naphthalen‐2‐yl)propan‐2‐ol (36.74 min), bisabolol (37.22 min), and 3,7,11,15‐tetramethyl‐2‐hexadecen‐1‐ol (40.18 min)). A steroidal compound, androstan‐3‐one, 17‐hydroxy‐1,17‐dimethyl‐ (47.49 min), was also identified.

**TABLE 3:** Phytochemicals present in *C. sativa* inflorescence methanol extract.

S/N	Retention time	Name of compound	Molecular weight	Fragment ion *m/z*	Formula
1	30.18	Caryophyllene	204	41,93,161,189,204	C_15_H_24_
2	33.84	7‐epi‐trans‐sesquisabinene hydrate	222	119,121,204,222	C_15_H_26_O
3	34.84	Caryophyllene oxide	220	41, 43,55,93,109,162,220	C_15_H_24_O
4	36.74	2‐(4a,8‐Dimethyl‐1,2,3,4,4a,5,6,8a‐octahydro‐2‐naphthalenyl)‐2‐propanol	222	59, 93,109,189,204,205,222	C_15_H_26_O
5	37.22	Bisabolol	222	119,121,122,204,222	C_15_H_26_O
6	40.18	3,7,11,15‐Tetramethyl‐2‐hexadecen‐1‐ol	296	81,95,124,137,279,296	C_20_H_40_O
7	43.28	Hexadecane, 1,1‐bis(dodecyloxy)‐	594	43,57,69,83,97,98,140,222,408,594	C_40_H_82_O_2_
8	43.66	Hexadecanoic acid, ethyl ester	284	88,101,157,284	C_18_H_36_O_2_
9	47.01	9,12‐Octadecadienoic acid, ethyl ester	308	67,81,95,96,110,263,308	C_20_H_36_O_2_
10	47.49	Androstan‐3‐one, 17‐hydroxy‐1,17‐dimethyl‐	318	55,56,81,175,215,259,284,318	C_21_H_34_O_2_
11	47.96	1‐Heptatriacotanol	536	55,69,79,82.97,123,140,269,459,536	C_37_H_76_O
12	49.98	8‐Tetrahydrocannabinol	314	55,69,107,231,314	C_21_H_30_O_2_
13	50.69	Tetrahydrocannabivarin	286	41,43,81,189,203,271,286	C_19_H_26_O_2_
14	51.81	Cannabivarin	282	41,238,267,282	C_19_H_22_O_2_
15	52.43	Cannabidiol	314	67,121,193,231,246,314	C_21_H_30_O_2_
16	54.44	9‐Tetrahydrocannabinol	314	55,91,115,128,231,271,299,314	C_21_H_30_O_2_
17	55.14	Cannabigerol	316	123,193,231,247,316	C_21_H_32_O_2_
18	55.51	Cannabinol	310	46,119,165,238,295,296,310	C_21_H_26_O_2_
19	56.73	8‐Hydroxy‐delta‐9‐THC	330	43,91,193,214,271,311,315,330	C_21_H_30_O_3_

*Note*: Compounds were tentatively identified using Thermo Xcalibur 4.0 software by comparing mass spectra with those in the 2017 NIST Mass Spectral Library, and the confidence level for molecular identification was based on NIST library match.

### 3.3 TLC Identification of Cannabinoids in the Extracts

**FIGURE 1:**
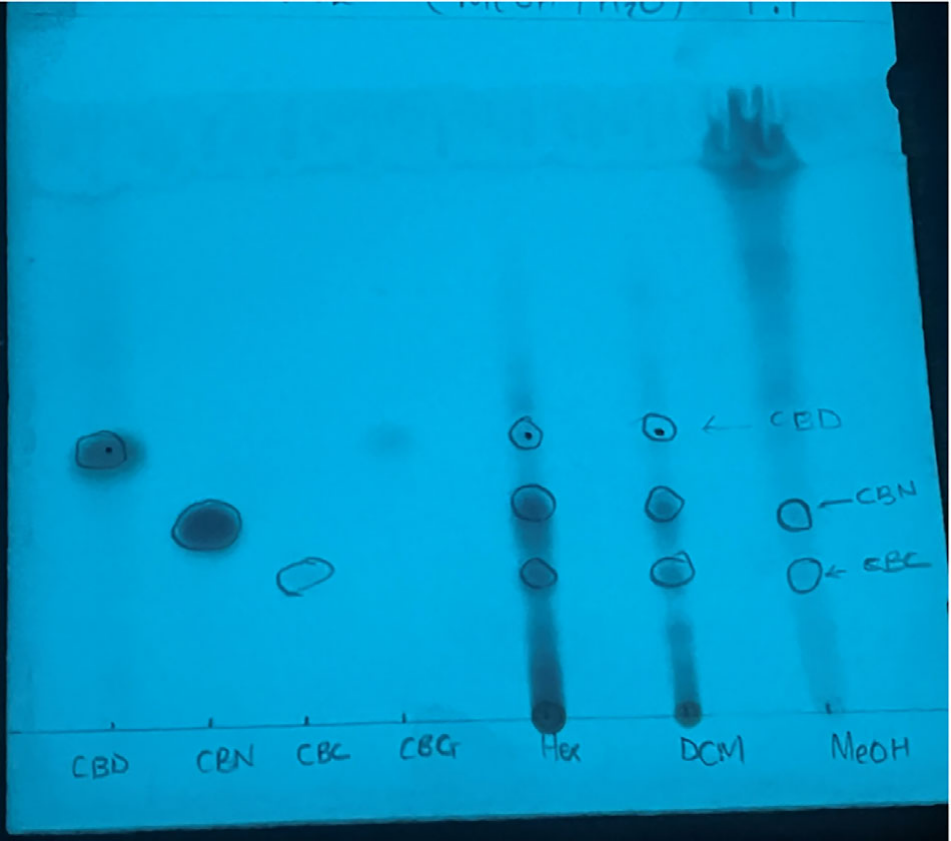
TLC analysis of hexane, dichloromethane, and methanol extracts showing the presence of CBD, CBN, and CBC.

TLC analysis detected CBD in the hexane and dichloromethane extracts (Rf = 0.40), CBN in the hexane, dichloromethane, and methanol extracts (Rf = 0.2875), and CBC in all three extracts (Rf = 0.20), with each Rf matching its respective reference standard (Figure 1).

### 3.4 Effect of *C. sativa* Inflorescence on α‐Amylase Enzyme Activity

As presented in Table 4, the different extracts (Hexane, dichloromethane, and methanol) inhibited α‐amylase enzyme at the various test concentrations. The inhibition was dose‐dependent on all the extracts tested. Hexane extract showed a mean percentage inhibition of 12.66 ± 0.26 at 62.5 µg/mL and 44.19 ± 0.19 at 500 µg/mL, with an IC_50_ of 727 µg/mL. The dichloromethane extract showed a mean percentage inhibition of 1.5±0.20 at 62.5 µg/mL and 14.65 ± 0.46 at 500 µg/mL, with an IC_50_ higher than 1000 µg/mL. The methanol extract showed a mean percentage inhibition of 1.46±0.04 at 62.5 µg/mL and 10.00 ± 0.39 at 500 µg/mL, with an IC_50_ higher than 1000 µg/mL. Among the extracts, the hexane extract demonstrated the highest α‐amylase activity, though less potent than the standard drug (acarbose), which showed a mean percentage inhibition of 13.39±0.43 at 12.5 µg/mL and 72.70 ± 1.03 at 100 µg/mL with an IC_50_ of 54.27 µg/mL.

**TABLE 4:** *C. sativa* inflorescence hexane, DCM, and methanol extracts percentage *α*‐amylase inhibition in comparison with acarbose standard drug.

Extracts	Concentrations (µg/mL)	% Inhibition (Mean ± SD)	IC_50_ (µg/mL)
Hexane	62.5	12.66 ± 0.26	727.57 ± 3.00
	125	20.03 ± 2.38	
	250	34.33 ± 0.66	
	500	44.19 ± 0.29	
DCM	62.5	1.50 ± 0.20	>1000
	125	4.36 ± 0.44	
	250	8.19 ± 0.54	
	500	14.65 ± 0.46	
Methanol	62.5	1.46 ± 0.04	>1000
	125	6.33 ± 0.23	
	250	7.14 ± 0.09	
	500	10.00 ± 0.39	
Acarbose **(Standard drug)**	12.5	13.39 ± 0.43	54.27 ± 0.01
	25	29.46 ± 0.47	
	50	38.26 ± 0.25	
	100	72.70 ± 1.03	

*Note*: Inhibition and IC_50_ values are presented as means ± SD (*n* = 3).

### 3.5 Effect of *C. sativa* Inflorescence on α‐Glucosidase Enzyme Activity

As displayed in Table 5, the hexane extract showed a mean percentage inhibition of 39.70 ± 0.03 at 62.5 µg/mL and 91.34 ± 0.52 at 500 µg/mL, with an IC_50_ of 99 µg/mL. The dichloromethane showed a mean percentage inhibition of 38.93 ± 0.83 at 62.5 µg/mL and 68.78 ± 1.05 at 500 µg/mL, with an IC_50_ of 169 µg/mL. The methanol showed a mean percentage inhibition of 72.55 ± 0.21 at 62.5 µg/mL and 96.44 ± 0.57 at 500 µg/mL, with an IC_50_ of 11.21 µg/mL. Among the extracts, the methanol extract demonstrated the strongest α‐glucosidase inhibitory effect, followed by the hexane extract, which also showed a notable effect.

**TABLE 5:** *C. sativa* inflorescence hexane, DCM, and methanol extracts percentage α‐glucosidase inhibition in comparison with the acarbose standard drug.

Extracts	Concentrations (µg/mL)	% Inhibition (Mean ±SD)	IC_50_ (µg/mL)
Hexane	62.5	39.70 ± 0.03	99.19 ± 0.53
	125	55.75 ± 0.09	
	250	69.39 ± 0.65	
	500	91.34 ± 0.52	
DCM	62.5	38.93 ± 0.83	169.00 ± 1.89
	125	41.27 ± 0.67	
	250	52.68 ± 0.40	
	500	68.78 ± 1.05	
Methanol	62.5	72.55 ± 0.21	11.21 ± 0.47
	125	75.26 ± 0.71	
	250	84.51 ± 0.34	
	500	96.44 ± 0.57	
Acarbose **(Standard drug)**	12.5	38.51 ± 0.53	22.07 ± 2.51
	25	47.06 ± 0.43	
	50	66.73 ± 0.78	
	100	77.05 ± 1.17	

*Note*: Inhibition and IC_50_ values are presented as means ± SD (*n* = 3).

### 3.6 Effect of *C. sativa* Inflorescence on Glucose Uptake in Yeast Cell Suspension

**FIGURE 2:**
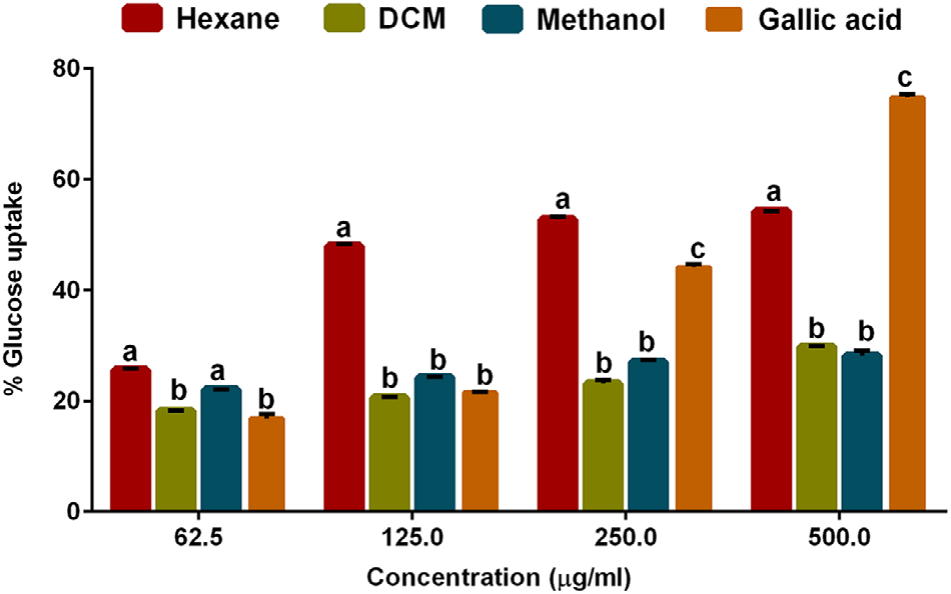
Percentage glucose uptake activities of *C. sativa* inflorescence hexane, DCM, and methanol extracts in comparison with the gallic acid standard. Each bar data represent mean ± SD of three separate experiments (*n* = 3). At each concentration, bars with different letters (a–c) show statistically significant differences at *p* < 0.05 using Tukey's multiple comparison post hoc test.

The results of the glucose uptake effect of the extract samples presented in Figure 2 showed a mean percentage of glucose uptake of 25% at 62.5 µg/mL and 54% at 500 µg/mL for the hexane extract. At this same concentration, the dichloromethane extract showed a mean percentage glucose uptake of 18% and 30%, respectively. Although there were no significant differences (*p* > 0.05) in the glucose uptake effects of the methanol and DCM extracts at 125–500 µg/mL, the hexane extract demonstrated a higher uptake capacity at these concentrations.

### 3.7 Free Radical Scavenging Effect of *C. sativa* Inflorescence

**FIGURE 3:**
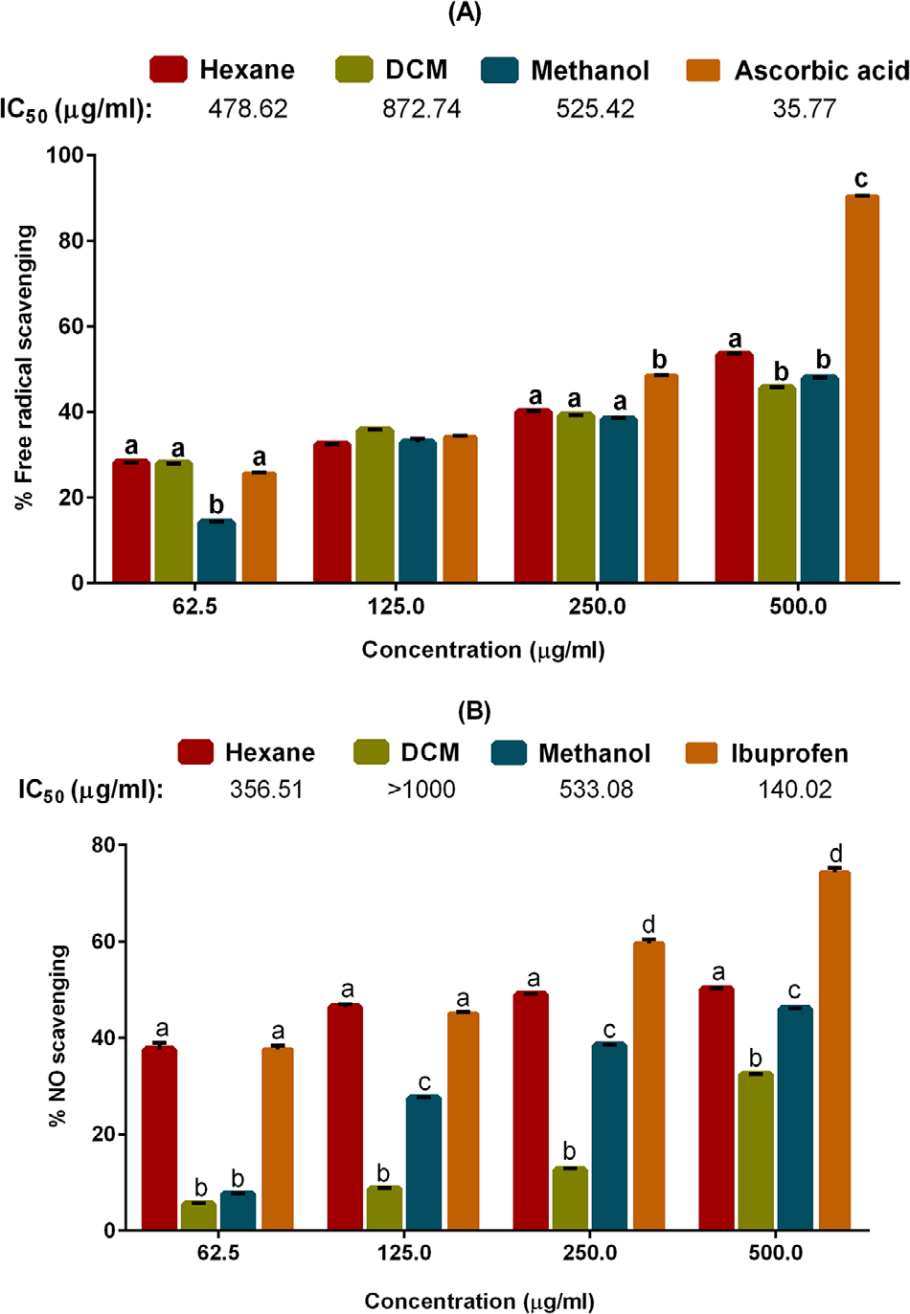
Percentage antioxidant activities of *C. sativa* inflorescence hexane, DCM, and methanol extracts in comparison with standard chemical compounds. Each bar data represent mean ± SD of three separate experiments (*n* = 3). At each concentration, bars with different letters (a–c) show statistically significant differences at *p* < 0.05 using Tukey's multiple comparison post hoc test.

In Figure 3A, the hexane extract's mean percentage free radical scavenging activity at 62.5 µg/mL and 500 µg/mL was 28% and 54%, respectively, producing an IC_50_ of 478 µg/mL. While the methanol extract activity (IC_50:_ 525 µg/mL) was statistically lower than that of the other extracts (62.5 µg/mL), there were no significant differences between the plant treatments at 125–250 µg/mL. Although the hexane extract demonstrated lower activity than the ascorbic standard (IC_50_: 35 µg/mL) at 500 µg/mL, it produced the best free‐radical scavenger activity among the tested plant samples.

### 3.8 Nitric Oxide Production Inhibitory Effect of *C. sativa* Inflorescence

Data in Figure 3B revealed the NO generation inhibitory capacity of the hexane, dichloromethane, and methanol extracts of *C. sativa* inflorescence. The hexane extract activity, which was 38% at 62.5 µg/mL, increased to 50% at 500 µg/mL. When compared with the ibuprofen with 70% activity at 500 µg/mL, the activities of the DCM and the methanol extracts were 62% and 38% lower than those of the standard chemical at the highest test concentration. With an IC_50_ of 356.51 µg/mL, the hexane extract showed the best NO‐production inhibitory capacity among the inflorescence extract samples.

### 3.9 Molecular Docking

**FIGURE 4:**
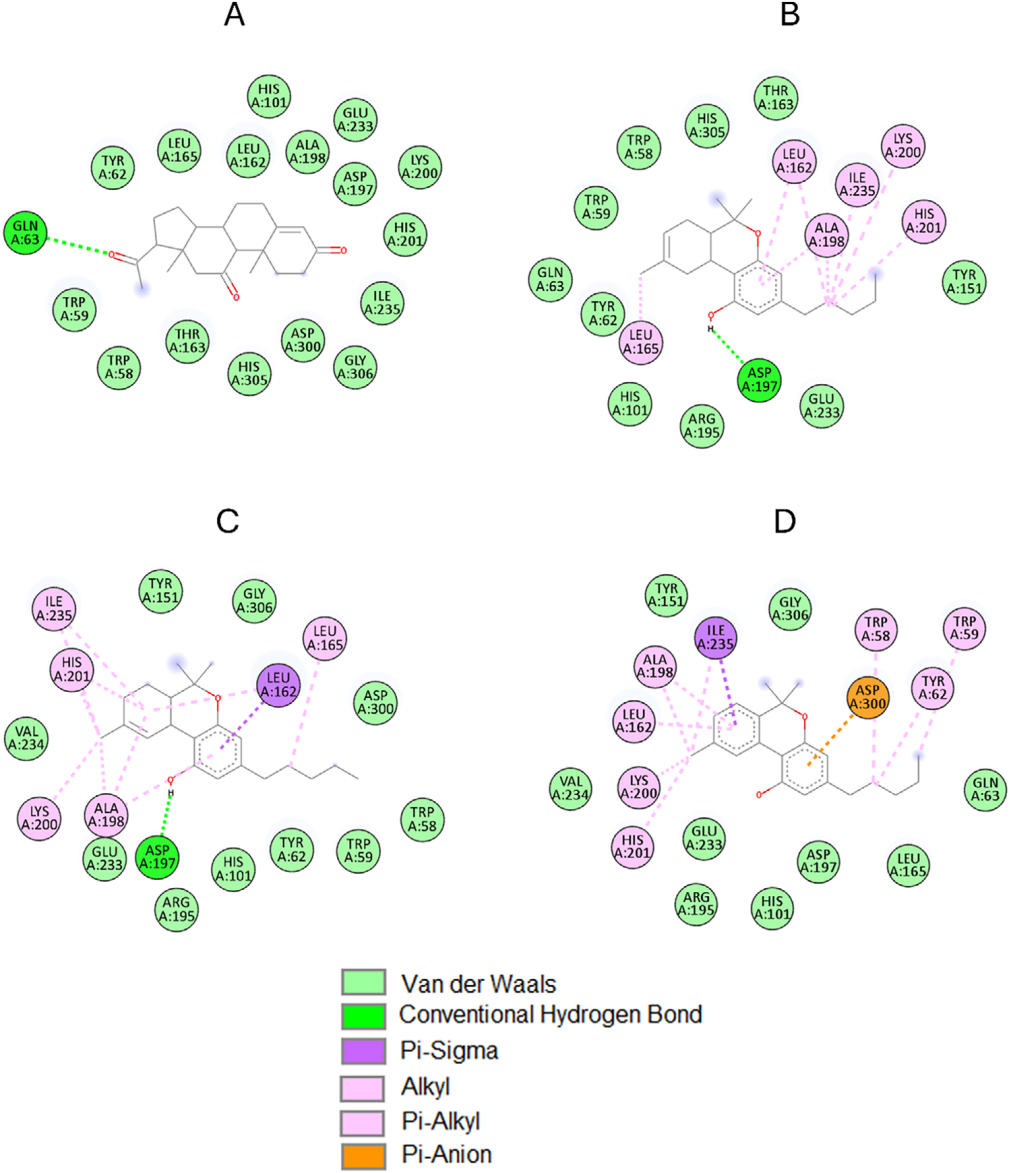
2D molecular interactions of *C. sativa* inflorescence: (A) 11‐ketoprogesterone, (B) 8‐tetrahydrocannabinol, (C) 9‐tetrahydrocannabinol, and (D) cannabinol with α‐amylase active site amino acid residues. Images generated using BIOVIA Discovery Studio V. 24.1.0.23298.

**FIGURE 5:**
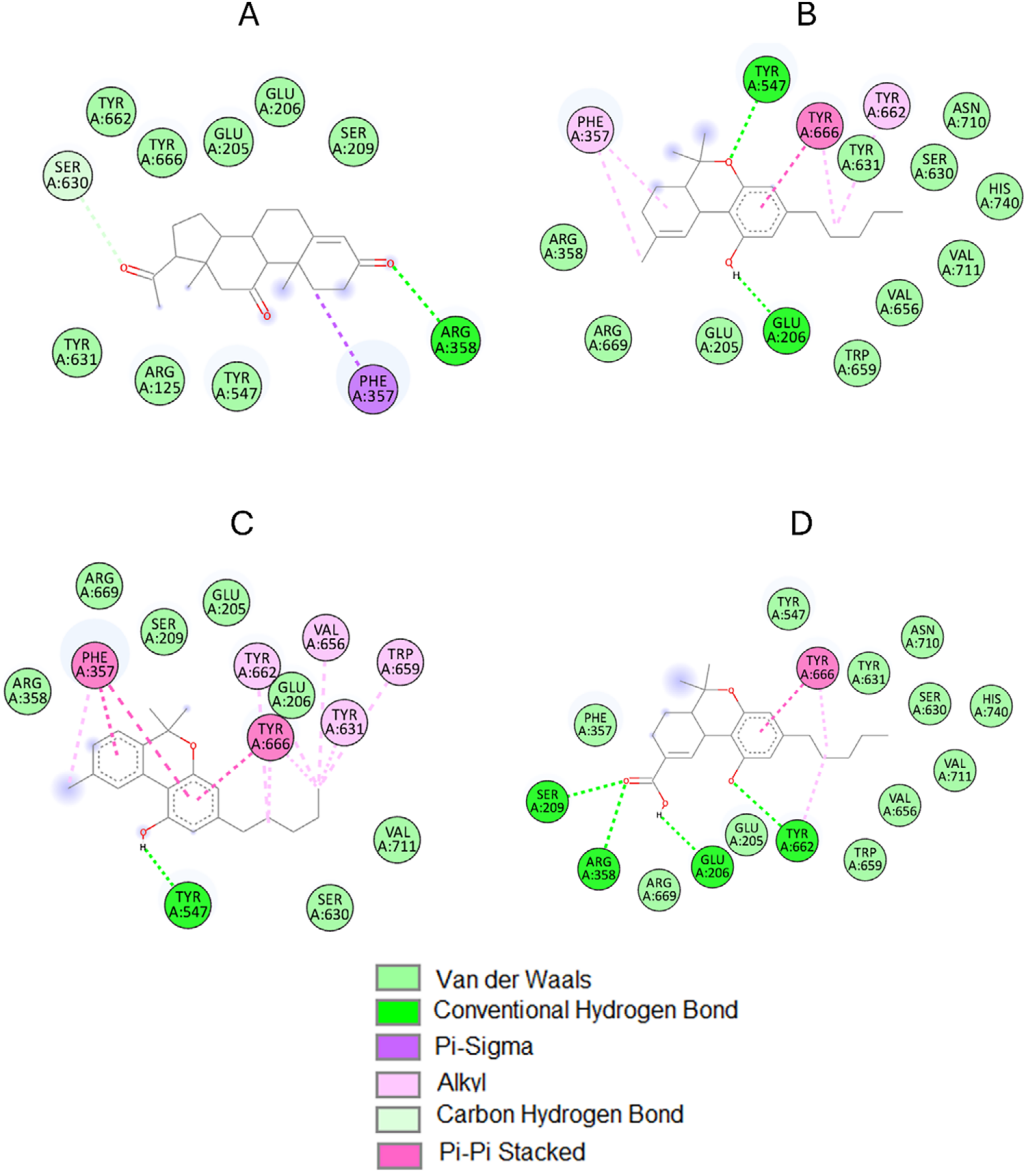
2D molecular interactions of *C. sativa* inflorescence: (A) 11‐ketoprogesterone, (B) 9‐tetrahydrocannabinol, (C) cannabinol, and (D) tetrahydrocannabinol‐7‐oic acid with DPP‐4 active site amino acid residues. Images generated using BIOVIA Discovery Studio V. 24.1.0.23298.

**FIGURE 6:**
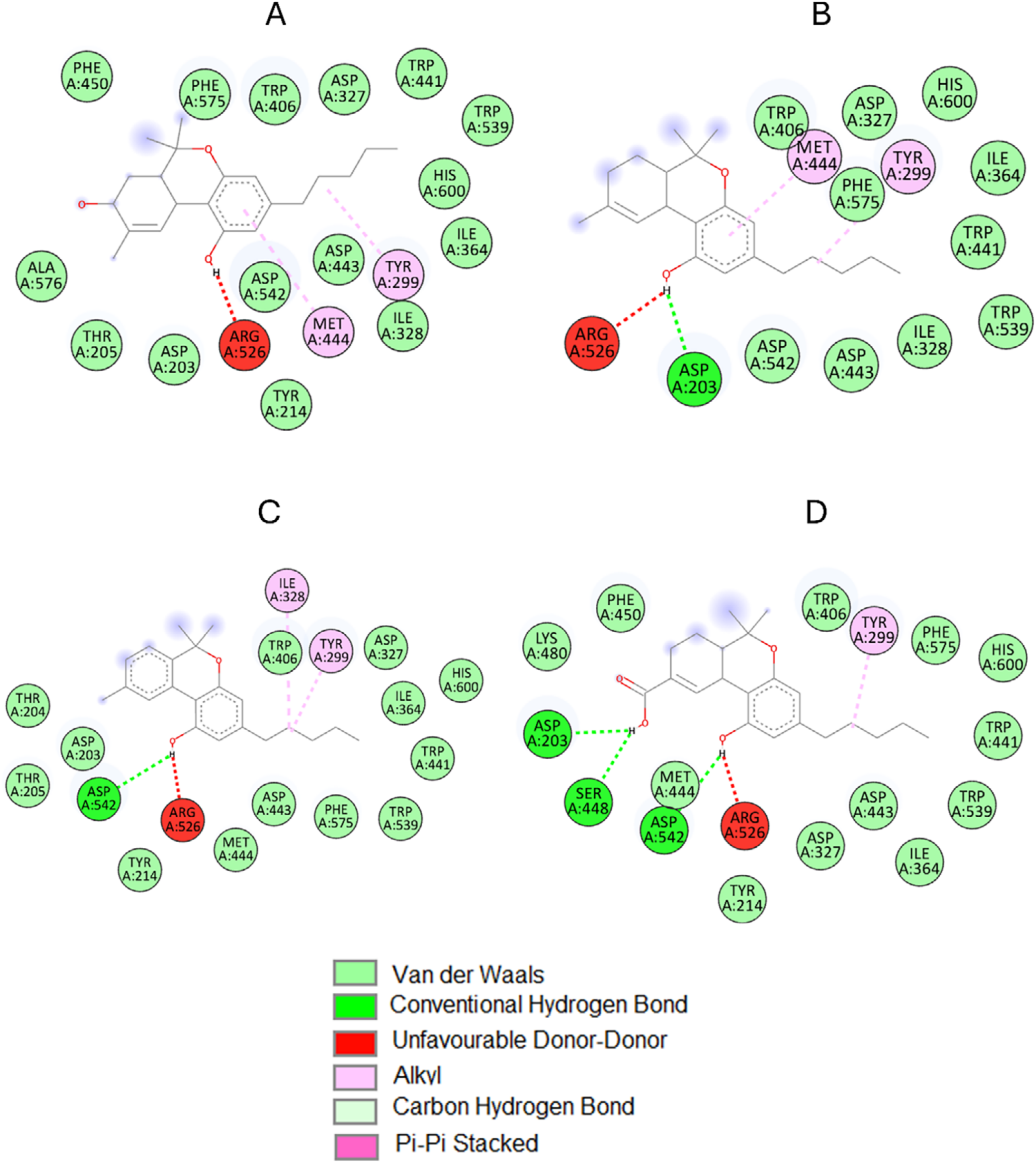
2D molecular interactions of *C. sativa*: (A) 8‐hydroxy‐delta‐9‐THC, (B) 9‐tetrahydrocannabinol, (C) cannabinol, and (D) tetrahydrocannabinol‐7‐oic with α‐glucosidase active site amino acid residues. Images generated using BIOVIA Discovery Studio V. 24.1.0.23298.

**FIGURE 7:**
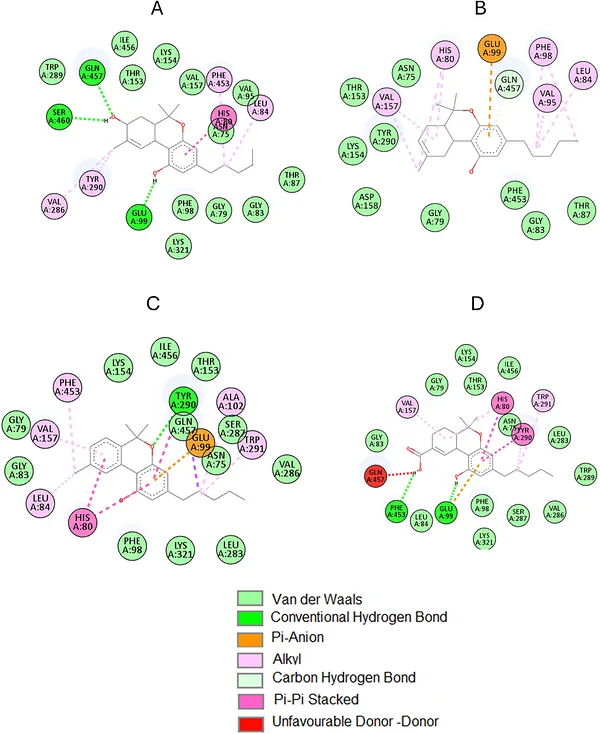
2D molecular interactions of *C. sativa*: (A) 8‐hydroxy‐delta‐9‐THC, (B) 8‐tetrahydrocannabinol, (C) cannabinol, and (D) tetrahydrocannabinol‐7‐oic acid with SGLT‐2 active site amino acid residues. Images generated using BIOVIA Discovery Studio V. 24.1.0.23298.

**FIGURE 8:**
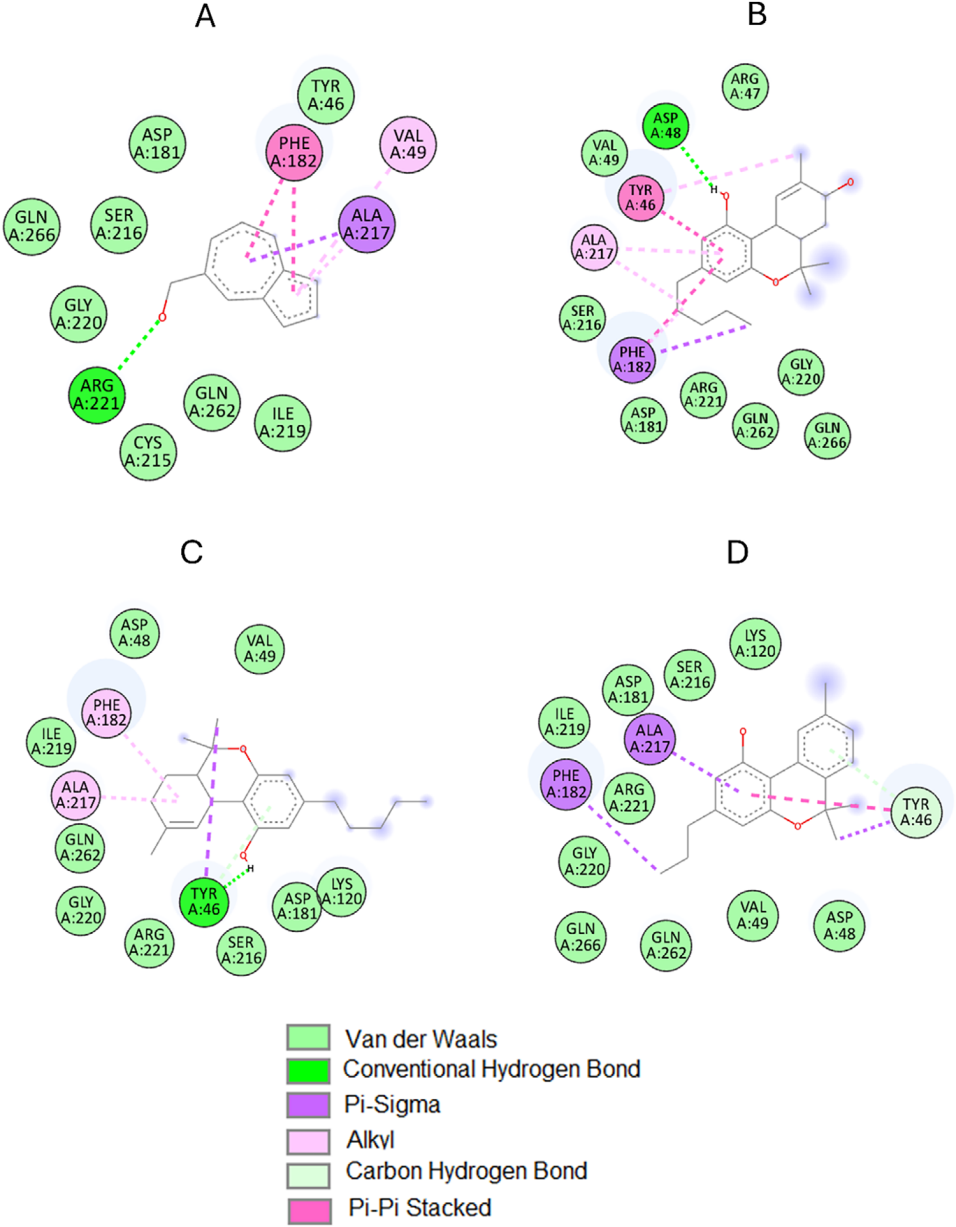
2D molecular interactions of *C. sativa*: (A) 5‐azulenemethanol, (B) 8‐hydroxy‐delta‐9‐THC, (C) 9‐tetrahydrocannabinol, (D) cannabivarin with PTP1B active site amino acid residues. Images generated using BIOVIA Discovery Studio V. 24.1.0.23298.

After docking the 36 compounds from the various *C. sativa* inflorescence with the 4 inhibitors of the target proteins, the results of ligand‐protein complexes with higher binding energy scores are presented in Table 6. Though there are computed binding scores for all the phytocompounds from the studied plant, those with scores less than −6.7, −6.2, −6.0, −7.2, and −8.0 kcal/mol with α‐amylase, DPP‐4, α‐glucosidase, SGLT‐2, and PTP1B, respectively, were excluded from the Table. When considering the affinity between acarbose and the two carbohydrate digestive enzymes investigated, both 9‐tetrahydrocannabinol (−8.7, −7.6 kcal/mol) and cannabinol (−8.6, −7.6 kcal/mol) had respective calculated lower scores than the standard antidiabetic drug. The binding energy of 9‐tetrahydrocannabinol with DPP‐4 was the same as that of sitagliptin (−9.1 kcal/mol), while the −9.8 kcal/mol for 8‐hydroxy‐delta‐9‐THC with SGLT‐2 was higher than −10.6 kcal/mol of empagliflozin, a clinical inhibitor of the latter co‐transporter. With a docking score of −8.5 kcal/mol, cannabivarin had the strongest affinity for PTP1B among the characterized inflorescence phytochemicals. Visual inspection of the molecular interactions responsible for maintaining the compound at the target protein active site regions, as displayed in Figures 3, 4, 5, 6, 7, revealed the involvement of various polar and nonpolar bonds. While a single hydrogen bond was involved in 11‐ketoprogesterone interacted with α‐amylase (Figure 4A), 2 hydrogen bonds with alkyl and pi–pi stacked bond were responsible for 9‐tetrahydrocannabinol interaction with DPP‐4 (Figure 5B). Tetrahydrocannabinol‐7‐oic acid interacted with ASP203, SER448, ASP542, ARG526, TYR229 of α‐glucosidase (Figure 6D) whereas in 8‐hydroxy‐delta‐9‐THC bonding with SGLT‐2 (Figure 6A), SER460, GLN457, GLU99, ASN75, PHE453, LEU84, VAL 286 and TYR290 were involved in bond formation Moreover, in Figure 8D, cannabivarin formed pi–sigma and pi–pi stacking interactions with the PHE182, ALA217, and TYR46 amino acid residues of PTP1B.

**TABLE 6:** Binding free energy scores (kcal/mol) for *C. sativa* inflorescence metabolites with diabetic protein targets.

	α‐Amylase	DPP‐4	α‐Glucosidase	SGLT‐2	PTP1B
1,1,4,4,7,7‐Hexamethyltrindan	−8.2	−7.9	−7.2	−8.2	−7.2
11‐Ketoprogesterone	−**9.7**	**−8.7**	−7.3	−9.0	−6.2
1‐Chloro‐3,6‐dimethoxycrinan‐2‐ol	−7.8	−8.0	−6.2	−8.5	−6.0
5‐Azulenemethanol	−6.7	−7.2	−7.1	−8.0	**−7.6**
8‐Hydroxy‐delta‐9‐THC	−7.8	−7.5	**−7.8**	**−9.8**	**−7.6**
8‐Tetrahydrocannabinol	−**8.5**	−8.6	−7.4	**−9.6**	−7.2
9‐Tetrahydrocannabinol	−**8.7**	**−9.1**	**−7.6**	−9.2	**−7.6**
Bisabolol	−7.2	−7.3	−6.9	−8.2	−6.7
Cannabidiol	−7.4	−7.3	−7.2	−9.2	−6.8
Cannabigerol	−7.2	−7.5	−7.0	−8.9	−6.8
Cannabinol	−**8.6**	**−8.8**	**−7.6**	**−9.6**	−7.5
Cannabivarin	−8.4	−8.4	−7.1	−9.3	**−8.5**
Eudesm‐4(14)‐en‐11‐ol	−8.0	−7.3	−6.5	−8.3	−6.3
Eudesma‐4(14),11‐diene	−7.8	−7.3	−6.5	−8.0	−6.5
Tetrahydrocannabinol‐7‐oic acid	−8.4	**−8.7**	**−7.9**	**−9.4**	−7.0
Sitagliptin*	—	−9.1	—	—	—
Acarbose*	−6.9	—	6.6	—	—
Empagliflozin*	—	—	—	−10.6	—
3,4‐Dibromo‐5‐(2‐bromo‐3,4‐dihydroxy‐6‐(isopropoxymethyl)benzyl)benzene‐1,2‐diol**^#^**	—	—	—	—	−5.6

* = Standard drugs; **#** = Reported inhibitor (Wang et al., 2015); Column bold scores = Four (4) compounds with the lowest binding energies with respective protein targets in each column.

## 4 Discussion

The present study demonstrated that different solvent extracts of *C. sativa* inflorescences possess potential antidiabetic activity, with significant inhibitory effects on α‐glucosidase and α‐amylase. Various solvents have been used to isolate pharmacologically active compounds from *C. sativa*. Some of these compounds include cannabinoid phenols, flavonoids, terpenoids, alcohols, aldehydes, *n*‐alkanes, wax esters, steroids, alkaloids, and non‐cannabinoid phenols such as stilbenoids, lignans, spiro‐indans, and dihydrophenanthrenes [11]. These compounds possess varying polarities, which influence their pharmacological activities. Phytochemical analysis of the various *C. sativa* inflorescence extracts in our current study revealed the presence of monoterpenes, such as limonene, sesquiterpenes, including caryophyllene, and other cannabinoids. In line with our findings, Kotiranta et al. [19] detected these terpenes together with the cannabinoids tetrahydrocannabivarin and cannabidiol in *C. sativa* inflorescence. Moreover, GC–MS analysis conducted by Calvi et al. [20] and Namdar et al. [21], also identified the presence of α‐humulene, limonene, caryophyllene oxide, nerolidol, and several other terpene derivatives in oils obtained from similar *C. sativa* samples. The results from the TLC analysis and the standards validated some of the findings from the GC–MS as presented in Figure 1. The similarity in Rf values and chromatographic characteristics between the sample bands and the reference standards supports the tentative identification of these compounds.

The different extracts of *C. sativa* exhibited remarkable inhibitory effects against α‐amylase and α‐glucosidase, as shown in Tables 4 and 5. Among the extracts, hexane showed the highest overall enzyme inhibitory effect, followed by dichloromethane and methanol. The observed activity of the hexane extract could be attributed to the presence of phytocannabinoids, which have previously been reported to inhibit key enzymes involved in glucose metabolism more effectively than those present in dichloromethane and methanol extracts. This inhibitory effect of the hexane extract of *C. sativa* corroborates previous reports indicating that hexane plant extracts contain bioactive compounds with significant inhibitory effects on insulin resistance, α‐amylase, and α‐glucosidase [22, 23, 24].

The differences in biological activity observed among the hexane, dichloromethane, and methanol extracts should be interpreted in the context of solvent‐dependent phytochemical selectivity. Sequential extraction with solvents of increasing polarity enriches different classes of metabolites because cannabinoids and many terpenoids are predominantly lipophilic and are preferentially extracted by nonpolar solvents such as hexane, whereas more polar solvents recover relatively higher proportions of polar constituents. Therefore, the superior activity observed for the hexane extract in some assays likely reflects differences in the phytochemical composition generated by solvent selectivity rather than an intrinsic superiority of the plant material. This interpretation is consistent with recent studies demonstrating that extraction solvent substantially influences cannabinoid recovery, metabolite profiles, and the resulting biological activities of *C. sativa* extracts [19].

The results of glucose uptake in yeast cells showed that the hexane extract exhibited the highest activity in enhancing glucose uptake, compared to the dichloromethane and methanol extracts (Figure 2). In this regard, phytochemicals have been reported to improve glucose uptake in body tissues through the modulation of glucose transporter proteins [14, 25, 26]. In line with previous investigations, the enhanced glucose uptake observed in yeast cells treated with the hexane extract may be attributed to phytocannabinoids modulating the microorganisms’ transporter proteins. However, in contrast to the present study, an enzymatic hydrolysate from *C. sativa* seed oil was reported to inhibit glucose transport in Caco‐2 cells more effectively than acarbose [27]. Although the yeast glucose uptake assay provides a useful preliminary screening model for identifying extracts with potential glucose‐utilization activity, it does not directly suggest similar insulin sensitization or enhanced glucose transporter modulation in an insulin‐responsive mammalian system. Nonetheless, further validation in mammalian cell models, such as adipocytes or skeletal muscle cells, as well as in vivo diabetic models, is required to validate these observations.

Oxidative stress and inflammation contribute to the development and progression of insulin resistance and β‐cell dysfunction. Although antioxidant compounds may help mitigate oxidative damage, the DPPH and NO assays employed in this study assess chemical radical production or scavenging capacity under defined in vitro conditions, as potential mechanisms that could protect tissues such as pancreatic β‐cells [4, 28]. Findings from this study showed that the hexane extract exhibited greater NO inhibitory activity and a stronger antioxidant effect than the other extracts (Figure 3). Sodium nitroprusside spontaneously releases NO under physiological conditions. After liberation, NO rapidly reacts with dissolved oxygen, generating nitrite ions that are detected by the Griess reagent. Even though normal levels of NO could play important roles in cellular signaling and vasodilation, abnormal levels of the messenger molecule could react with superoxide radical to produce extremely reactive peroxynitrite (ONOO^−^) radical. Consequently, maintaining the cellular concentration of NO at physiological levels is important for optimal biological function. In this regard, antioxidant constituents such as phenolic cannabinoids and oxygenated terpenoids, which could intercept NO or reactive nitrogen intermediates before nitrite formation, may have protective effects in disease conditions, including chronic inflammation linked to abnormal levels of the chemical. The observed activity of the cannabis extracts in this study may be attributed to oxygenated terpenoids, cannabinoids, and other redox‐active phytochemicals. However, this proposed mechanism may not fully account for the antioxidant action of this botanical product in vivo, as factors such as cellular specificity and phytochemical solubility may also influence its therapeutic efficacy.

Over the last decade, molecular docking computational analysis has emerged as a helpful tool employed in drug discovery. This computer‐aided method used to screen libraries of chemical compounds for druggable molecules in neurodegenerative, inflammatory, and cancer pathophysiology has been described as saving time and reducing costs associated with conventional in vitro and animal experiments [29, 30]. The results obtained after screening compounds from the *C. sativa* inflorescence against selected diabetes‐related protein targets revealed that some *C. sativa* phytoconstituents could interact favorably with these targets in a manner similar to some clinically approved standard drugs. For instance, chemical inhibitors of DPP‐4 and PTP1B improve insulin signaling in type 2 diabetes, whereas SGLT‐2 inhibitors such as empagliflozin enhance glucose excretion in the kidney. Based on this study outcome, 8‐hydroxy‐Δ^9^‐THC, Δ^9^‐tetrahydrocannabinol, cannabivarin, and tetrahydrocannabinol‐7‐oic acid demonstrated stronger binding affinities than the investigated standard drugs. For a chemical reaction to occur, reacting molecules must come in close proximity. Stronger binding among the phytoconstituents could mediate interactions that more effectively modulate the plant's inhibitory capacity against diabetic protein targets. Although this observed docking result suggests a proposed mechanism of action, it does not confirm the plant's antidiabetic therapeutic efficacy. Establishing these pharmacological properties will require further validation using complementary computational approaches, such as molecular dynamics simulations together with other biochemical and in vivo studies.

Despite the promising outcomes from this work, the study is not without limitations. The phytochemical characterization of the *C. sativa* extracts was based primarily on qualitative GC–MS analysis and library matching. Although this approach is widely used for phytochemical profiling, structurally related cannabinoids, particularly positional isomers and oxidized derivatives, may exhibit similar fragmentation patterns, making unequivocal compound identification challenging. Moreover, the GC–MS analysis was qualitative rather than quantitative, and the concentrations of the identified phytoconstituents were not determined. As a result, direct correlations between phytochemical composition and the observed biological activities could not be established. Another limitation of the present study is that the antioxidant activity was evaluated using chemical assays (DPPH scavenging and NO inhibition), which measure radical‐quenching capacity under assay‐specific conditions. While these assays provide useful preliminary information on the redox properties of the extracts, they do not necessarily predict biological antioxidant activity or therapeutic efficacy in vivo. Consequently, Further investigations using cell‐based systems and animal models are required to determine whether these extracts modulate oxidative stress pathways relevant to diabetes.

## 5 Conclusion

This study provides preliminary evidence that *C. sativa* inflorescence extracts contain phytoconstituents with antioxidant, enzyme‐inhibitory, and potential antidiabetic activities under the experimental conditions investigated. Among the extracts, the hexane extract exhibited the strongest enzyme inhibition and capacity to interact with free radicals. However, further studies are required to determine whether its antioxidant activity contributes to its antidiabetic effects in vivo. In addition, compounds including Δ^9^‐tetrahydrocannabinol, 8‐hydroxy‐Δ^9^‐THC, cannabivarin, and tetrahydrocannabinol‐7‐oic acid exhibited favorable binding affinities toward diabetes‐related protein targets, identifying them as promising candidates for further investigation. Nevertheless, these molecular docking findings are exploratory and require further confirmation through advanced phytochemical characterization, biochemical assays, mechanistic studies, and in vivo investigations before any therapeutic potential can be assertively established.

## Author Contributions

Conceptualization: R.M.C, V.L.N.N., and I.A.; methodology: R.M.C, V.L.N.N, I.A., K.O., and TJ.F.N.T.; validation: R.M.C, V.L.N.N., I.A., and K.O.; formal analysis, R.M.C, V.L.N.N., I.A., and K.O.; data curation R.M.C, V.L.N.N., I.A., and K.O.; initial draft preparation R.M.C, V.L.N.N., and I.A.; review and editing, all authors; funding, M.G.M., supervision, M.G.M. All authors have read and agreed to the published version of the manuscript .

## Conflicts of Interest

The authors declare no conflicts of interest.

## Data Availability

The data supporting the findings of this study are available within the article and its Supporting Information. Additional data are available from the corresponding author upon request.
